# Effect of Ti Doping on the Grain Boundary Phases in Sintered Nd-Ce-Fe-B and Its Influence on the Diffusion Behavior of Heavy Rare Earth Dy

**DOI:** 10.3390/ma19050916

**Published:** 2026-02-27

**Authors:** Lisheng Ye, Huanmao Yao, Quan Fang, Tongxiang Liang, Lei Wang

**Affiliations:** 1 School of Materials Science and Engineering, Jiangxi University of Science and Technology, Ganzhou 341000, China; 2 School of Material Science and Engineering, Tiangong University, Tianjin 300387, China; 3 Jiangxi Province Key Laboratory of Magnetic Metallic Materials and Devices, Ganzhou 341000, China

**Keywords:** grain boundary diffusion, Dy distribution, coercivity, Ti doping, RE_6_Fe_13_Ga

## Abstract

This study addresses the issue of rare earth (RE) resource wastage caused by the aggregation of the commonly used diffusion source, Dy, at the triangular grain boundary region during grain boundary diffusion (GBD). The approach involves Ti doping to refine the grain size and increase the volume fraction of RE_6_Fe_13_Ga, thereby improving the efficiency of Dy utilization. The results show that when 0.2 wt% Ti is doped, Dy diffusion is applied to the magnet, and the magnet achieves excellent magnetic properties, with Br = 14.03 kGs, Hcj = 20.24 kOe, Q = 0.96, and (BH)max = 47.15 MGOe. The coercivity shows an enhancement of 8.66 kOe compared to the pristine magnet. Research and analysis indicate that doping Ti into the magnet promotes the formation of the RE6Fe13Ga phase, leading to the creation of continuous thin grain boundaries that weaken the exchange coupling between adjacent grains. Additionally, the presence of RE_6_Fe_13_Ga suppresses the segregation of Dy in the RE-rich phases, encouraging its further incorporation into the main phase and improving Dy utilization. This study demonstrates that appropriate Ti doping can effectively optimize Dy distribution within the magnet, reduce its aggregation in the triangular grain boundary region, and promote its incorporation into the main phase. This significantly reduces the amount of Dy required and provides a feasible approach to enhancing the efficiency of heavy rare earth resource utilization, thereby offering a path to the design of high-performance GBD magnets.

## 1. Introduction

Sintered Nd-Fe-B magnets are widely used in fields such as motors, wind power, and automobiles due to their High coercivity and High remanence. With the rapid development of the industry, medium and heavy rare earth resources such as Pr, Nd, Dy, and Tb have been rapidly consumed [1,2,3,4]. Replacing part of Nd with the more abundant Ce can significantly reduce the cost of the magnets [5]. However, the intrinsic magnetic properties of Ce-Fe-B are relatively low, and as the amount of Ce increases, the magnet’s performance deteriorates significantly. In order to improve the magnetic properties of Ce-containing magnets, researchers have conducted extensive studies on Ce-containing magnets [6,7,8,9]. The double-phase method has been used to prepare Ce-containing Nd-Fe-B magnets [10], resulting in a significant improvement in the performance of the magnets. GBD is an effective method for enhancing the coercivity of magnets. GBD allows heavy rare earth elements (HREs) to form a magnetic hardening shell structure along the surface of the matrix, thereby improving coercivity. This technique allows for a significant improvement in coercivity while minimizing the loss of remanence when only small amounts of heavy rare earths are added to the magnet [11,12,13,14,15].

In recent years, the combination of GBD technology with Ce-containing Nd-Fe-B magnets has gained increasing attention. Numerous researchers have significantly enhanced the diffusion efficiency of heavy rare earth elements in magnets by optimizing the composition of diffusion sources, such as DyF3 [16], Nd-Dy-Al [17], Pr-Tb-Cu [4], and Gd-Y-Cu-Al [18]. This optimization has promoted the effective infiltration of these elements into the main phase, thereby improving the magnetic properties. However, current research on the influence of matrix composition on grain boundary diffusion behavior remains relatively limited. For example, Zhang et al. [19] conducted diffusion experiments on matrices with different main-phase compositions and pointed out that smooth and continuous grain boundary phases are beneficial for enhancing diffusion efficiency, thereby further optimizing the magnet’s performance. These studies mainly focus on improving the diffusion efficiency of rare earth elements, leading to significant achievements in the fabrication of high-performance magnets.

Currently, with the widespread application of grain boundary diffusion technology in industrial production, heavy rare earth elements are being rapidly consumed. Therefore, systematic research on the rational distribution and regulation of heavy rare earth elements within magnets is not only crucial for further optimizing coercivity but also holds significant importance for reducing production costs and achieving efficient resource utilization. Sasaki et al. [20] reported the mechanism of Ga in Nd-Fe-B magnets, indicating that the formation of the non-ferromagnetic RE_6_Fe_13_Ga phase effectively weakens the exchange coupling between the grains, thereby enhancing the coercivity of the magnet. Cui et al. [21] combined experimental methods with first-principles calculations to reveal that the affinity of the RE_6_Fe_13_Ga phase for Dy is relatively low, which leads to the preferential incorporation of Dy into the main phase. These studies provide valuable theoretical insights into the role of the RE_6_Fe_13_Ga phase in magnets. However, the specific mechanism of the influence of RE_6_Fe_13_Ga on Dy diffusion behavior, especially how the content of this phase affects the distribution of Dy, has not yet been fully understood. Ti has been reported to promote the formation of the RE_6_Fe_13_Ga phase and to improve the coercivity of the magnets [22]. However, research on the mechanism of Ti’s role in grain boundary diffusion is still relatively lacking. Therefore, this study systematically investigates the effect of Ti content on the microstructure and magnetic properties of Dy-diffused magnets, providing a theoretical basis for the optimization of diffusion processes and magnet design.

To further clarify the influence of RE_6_Fe_13_Ga on the grain boundary process of Dy, this work adjusts the Ti content in Ce-containing Nd-Fe-B magnets to regulate the RE_6_Fe_13_Ga phase in the matrix. First, magnets with different Ti contents were studied to elucidate the impact of Ti on their magnetic properties and microstructure. Based on these results, Dy diffusion was performed on magnets with different Ti contents. By analyzing the magnetic properties and microstructure of the magnets, the role of Ti in Dy diffusion was clarified, revealing how the addition of Ti enhances the utilization of Dy. This study has significant implications for the development of cost-effective, high-performance magnets.

## 2. Experiment

The materials were prepared using induction melting, melt spinning, hydrogen decrepitation, and air jet milling methods. The nominal composition of the magnets was (Pr_25_Nd_75_)_28_Ce_1.5_Fe_bal_(CuGaAl)_0.92_Ti_x_B_0.95_ (x = 0, 0.2, 0.4, 0.6 wt%). Grain size is D50 = 3.8–4.2 μm, and the particle shape is spherical. These powders were then placed in a press under a magnetic field of 1.5 T for alignment, forming square-shaped compacted billets. The billets were then subjected to isostatic pressing at 165 MPa. Finally, the green compact was sintered in vacuum at 1070 °C (1.0 × 10^−3^ Pa) for 8 h and then naturally cooled to room temperature. Subsequently, it was annealed at 900 °C for 3 h and naturally cooled to room temperature. Finally, a secondary annealing treatment was conducted at 440 °C for 3 h, followed by natural cooling to room temperature. The magnets were labeled as 0 Ti, 0.2 Ti, 0.4 Ti, and 0.6 Ti based on the Ti content.

The prepared matrices were sliced along the alignment direction into dimensions of 20 × 20 × 3 mm to serve as the substrates for diffusion. Pure Dy was used as the diffusion source. Thin disks of Dy metal, with a thickness of 0.5 mm, were cut using a wire cutting machine and placed on both poles of the magnet, perpendicular to the magnet’s surface. The diffusion treatment was performed under vacuum. Diffusion was carried out at 900 °C for 6 h and at 440 °C for 3 h. The magnets were labeled as 0 Ti GBD, 0.2 Ti GBD, 0.4 Ti GBD, and 0.6 Ti GBD based on the Ti content.

The magnetic properties of the magnets were measured at room temperature using a closed-loop permanent magnet measurement system (NIM-200C). X-ray diffraction (XRD, X PERT PRO, PANalytical, Alemlo, The Netherlands) with a Co target was employed to determine the phases present in the magnets. The element distribution and microstructure of the samples were observed using a field emission electron probe microanalyzer (EPMA, JXA-8350F, JEOL, Tokyo, Japan), a scanning electron microscope (SEM, S-3400N, Hitachi, Tokyo, Japan), and an energy-dispersive X-ray spectrometer (EDS), Transmission Electron Microscope (TEM, FEI-TALOS-F200X, FEI, Hillsboro, OR, USA).

## 3. Results and Discussion

**Figure 1:**
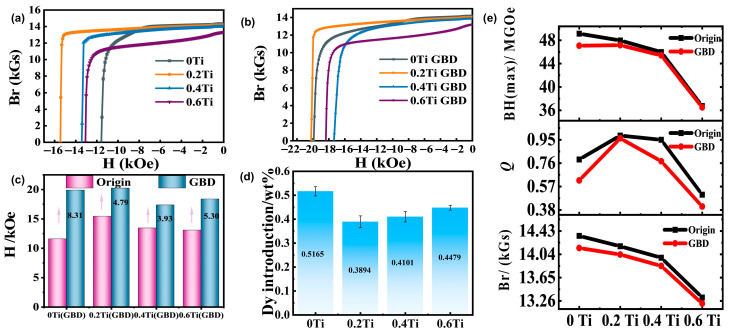
(**a**) Demagnetization curves after annealing for 0 Ti, 0.2 Ti, 0.4 Ti, and 0.6 Ti samples; (**b**) Demagnetization curves after annealing for 0 Ti GBD, 0.2 Ti GBD, 0.4 Ti GBD, and 0.6 Ti GBD samples; (**c**) Hcj and ∆Hcj of the magnets before and after GBD; (**d**) Average Dy content in the magnets after GBD; (**e**) (BH)max, Q, and Br of the original and GBD-treated magnets as a function of Ti content.

Figure 1a shows the second quadrant of the demagnetization curves (J-H curves) for magnets with different Ti contents. The results indicate that when the Ti content is 0.2 wt%, the intrinsic coercivity of the magnet increases from 11.57 kOe to 15.44 kOe. However, further increasing the Ti content leads to a decrease in coercivity. Figure 1b presents the second quadrant of the demagnetization curves for magnets with different Ti contents after grain boundary diffusion (GBD) treatment. Among them, the 0.2 Ti GBD sample exhibits the highest intrinsic coercivity, while the GBD sample without Ti (0 Ti GBD) has a slightly lower coercivity than the former.

To further investigate the correlation between the changes in magnetic properties before and after grain boundary diffusion, Figure 1e compares the variations in the maximum magnetic energy product, squareness, and remanence for magnets with different Ti contents before and after diffusion, with the corresponding magnetic performance parameters listed in Table 1. From the data in Figure 1e and Table 1 (The standard deviation of the magnetic properties is less than 5%), it can be observed that the remanence of the non-diffused magnets (0 Ti, 0.2 Ti, 0.4 Ti, 0.6 Ti) is 14.34 kGs, 14.17 kGs, 13.89 kGs, and 13.31 kGs, respectively, showing a gradual decrease with increasing Ti content. The maximum magnetic energy product follows a similar trend. The squareness values are 0.79, 0.98, 0.95, and 0.50, demonstrating an initial increase followed by a decrease. After Dy grain boundary diffusion, the remanence values of the magnets are 14.14 kGs, 14.03 kGs, 13.84 kGs, and 13.21 kGs, with squareness values of 0.62, 0.96, 0.77, and 0.40, respectively. The remanence only shows slight changes after diffusion, mainly due to Dy entering the main phase, which reduces the saturation magnetization. Squareness decreases slightly in the 0.2 Ti sample, while the other samples show varying degrees of deterioration. These results indicate that the Ti content significantly affects the diffusion behavior of Dy. Figure 1c further demonstrates the variation in coercivity increment as a function of Ti content. The coercivity increments for the 0 Ti, 0.2 Ti, 0.4 Ti, and 0.6 Ti samples are 8.31 kOe, 4.79 kOe, 3.93 kOe, and 5.30 kOe, respectively. Clearly, the coercivity of the magnet without Ti addition shows the most significant improvement. This is because the coercivity of the Ti-free sample is relatively low, making it easier to achieve a larger increase in coercivity. Additionally, the actual Dy content introduced in the 0.2 Ti magnet is the lowest, as shown in Figure 1d, while the Ti-free sample has the highest Dy content, reaching 5.1617 wt‰. It can be observed that the 0.2 Ti sample achieves the highest coercivity (Figure 1a) while introducing the lowest Dy content. This indicates that adding an appropriate amount of Ti can enhance the utilization of Dy.

**Figure 2:**
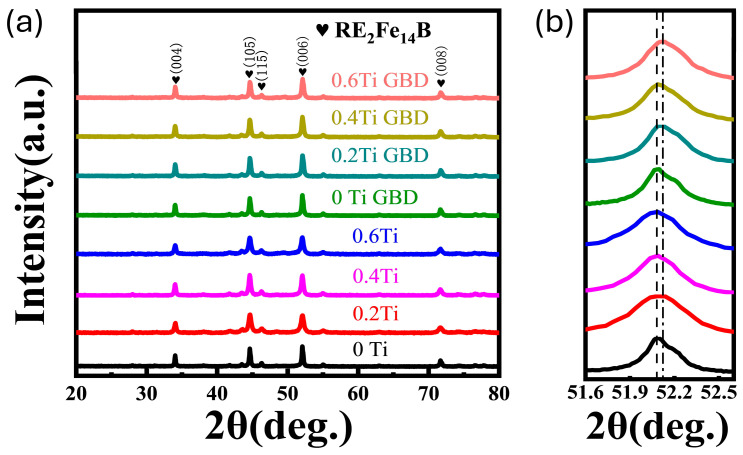
(**a**) XRD spectra of the original and GBD-treated magnets; (**b**) XRD spectra in the range of diffraction angles from 51.6° to 52.6° in (**b**).

Figure 2a shows the XRD spectra of magnets with different Ti contents before and after grain boundary diffusion. The diffraction peaks primarily correspond to the 2:14:1 main phase [23], and there is no significant shift in the main peaks of the magnets with different Ti contents, indicating that Ti does not enter the main phase. After Dy diffusion, the characteristic peak (006) of the main-phase shifts to a higher angle compared to the original magnet, as shown in Figure 2b. Based on the Bragg equation 2dsin θ = nλ, the shift in the diffraction peak to a higher angle indicates a decrease in the interplanar spacing [24]. Thus, this change in the characteristic peak can be attributed to the GBD process, where Dy enters the main phase and forms a (Nd, Dy)_2_Fe_14_B phase with a lower lattice constant in the main phase.

**Figure 3:**
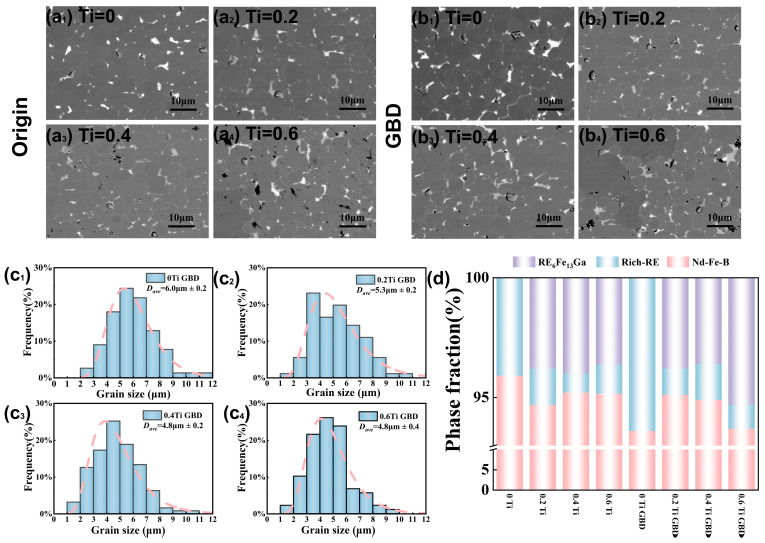
(**a_1_**–**a_4_**) SEM images of 0 Ti, 0.2 Ti, 0.4 Ti, and 0.6 Ti samples; (**b_1_**–**b_4_**) SEM images of 0 Ti GBD, 0.2 Ti GBD, 0.4 Ti GBD, and 0.6 Ti GBD samples; (**c_1_**–**c_4_**) Grain size distribution of 0 Ti GBD, 0.2 Ti GBD, 0.4 Ti GBD, and 0.6 Ti GBD samples; (**d**) Volume fraction diagrams of the grain boundary phase and main phase before and after diffusion.

The microstructure of a magnet is closely related to its magnetic properties. Figure 3 shows the microstructure of Ti-containing magnets before and after diffusion treatment. Specifically, Figure 3(a_1_–a_4_) corresponds to the microstructure of samples with Ti contents of 0, 0.2, 0.4, and 0.6 wt% before diffusion, while Figure 3(b_1_–b_4_) shows the corresponding microstructure after diffusion. By comparing the microstructures of the magnets with different Ti contents before diffusion, it can be observed that in the sample without Ti, the rich rare earth phase mainly accumulates at the triangular grain boundaries, leading to a lack of a continuous distribution of the rare earth grain boundary phase between the main phase grains. This results in incomplete magnetic isolation between the grains of the main phase, which in turn leads to lower coercivity. With the addition of Ti, the grain boundary phase gradually becomes more defined. As shown in Figure 3d, after adding Ti, the volume fraction of the grain boundary phase increases to varying degrees in the magnets. The grain boundary phase changes from a single white phase to a coexistence of white and gray phases. To confirm the main components of the grain boundary phase, semi-quantitative analysis was performed using energy dispersive X-ray spectroscopy (EDS). From Table 2, it can be observed that the white grain boundary phase has the composition of Nd_49.5_Pr_28.3_Ce_8_Fe_8.6_Ga_1_Cu_4.6_ (wt%), with a high rare earth content and low iron content, and is considered a rare earth-rich grain boundary phase. The gray grain boundary phase has the main composition of Pr_15.1_Nd_34.1_Ce_4.4_Fe_42.2_Ga_4.2_ (wt%). It is considered to be a Ga-rich grain boundary phase, and its composition is close to that of the RE_6_Fe_13_Ga phase. Formation of the gray phase increases the volume fraction of the grain boundary phase in the material, promoting the formation of continuous, thin-layered grain boundary structures. This, in turn, better encloses the main-phase grains, weakening the ferromagnetic coupling between the grains, which contributes to the significant improvement in coercivity [22]. However, when the Ti content is further increased, a noticeable decrease in coercivity occurs. The main reason for this is the increased volume fraction of the gray phase, which hinders the complete formation of the main-phase grains [25].

The EDS semi-quantitative analysis of the main-phase grains in the diffused magnets is shown in Table 2. It can be observed that after grain boundary diffusion, Dy elements are present in the main-phase grains, indicating that Dy has successfully diffused into the main phase and formed the (Pr, Nd, Ce, Dy)_2_Fe_14_B phase with higher magnetic anisotropy. This explains why the coercivity of the diffused magnets has increased. By comparing the microstructures before and after diffusion, it can be seen that in the sample without Ti, the grain boundary phase distribution becomes significantly more continuous after diffusion. As shown in Figure 3d, the volume fraction of the grain boundary phase in the 0 Ti sample increases greatly after diffusion, while the sample with 0.2 Ti shows no significant change in the volume fraction of the grain boundary phase after diffusion. This may be one of the reasons why the coercivity of the 0 Ti sample shows a larger increase after diffusion.

Figure 3(c_1_–c_4_) shows the grain size distribution of the samples after diffusion. It can be observed that the addition of Ti causes a significant change in the grain size. The grain size distribution of the diffused magnet without Ti is relatively broad, with an average size of approximately 6.0 μm. After adding 0.2 wt% Ti, the average grain size decreases to about 5.3 μm. As the Ti content continues to increase, the grain size becomes further refined. Based on the nucleation field model, the coercivity H_cj_ of the magnet can be expressed as [26]


(1) $$H_{\mathrm{cj}}=\alpha H_{A}\;-\;N_{\mathrm{eff}}M_{s}$$

In this equation, α represents the coefficient of the anisotropy field, which is influenced by defects in the magnet. $H_{A}$ is the anisotropy field, $M_{s}$ is the saturation magnetization, and $N_{\mathrm{eff}}$ is the demagnetizing factor, which is mainly determined by the grain size and shape, defects in the grain boundaries, and regions with antiferromagnetic domains. The results show that the addition of Ti can refine the RE_2_Fe_14_B main-phase grains, reduce the demagnetizing factor, and thus improve the coercivity of the magnet. However, when the Ti content reaches 0.6 wt%, abnormally large grains appear in the magnet. EDS analysis was conducted on the normal grains and the abnormally large grains in this sample, as shown for the 0.6 Ti GBD in Table 2. It was found that the Fe content in the abnormally large grains was significantly higher than that in the normal grains, and the BSE contrast for the normal grains was also inconsistent. This suggests that the formation of Nd_x_Fe_y_ may have occurred. Studies have shown that Ti has a stronger affinity for B than for Fe, which leads to Ti preferentially combining with B [27]. This results in a lack of B in the magnet, with insufficient B to combine with RE and Fe to form the main phase, leading to the formation of Nd_x_Fe_y_ phase and rare earth-rich phases [22]. This explains why the volume fraction of the grain boundary phase in the 0.6 Ti sample is significantly higher than in the other samples. The presence of abnormal phases in the 0.6Ti GBD sample led to significant degradation of the microstructure of the magnet, resulting in uneven distribution of different phases. This, in turn, adversely affected the squareness of the magnet, causing a substantial decline.

**Figure 4:**
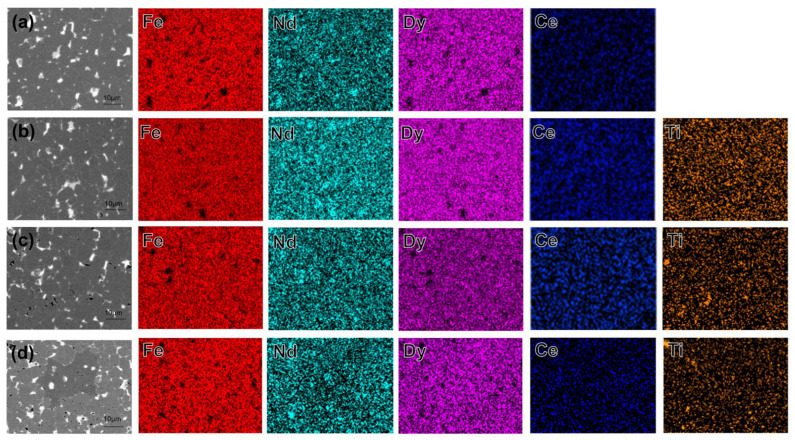
(**a**–**d**) are the EDS mapping images of the 0 Ti GBD, 0.2 Ti GBD, 0.4 Ti GBD, and 0.6 Ti GBD samples, respectively.

To investigate the element distribution in the diffused magnets, energy dispersive spectroscopy (EDS) analysis was conducted on the 0 Ti, 0.2 Ti, 0.4 Ti, and 0.6 Ti magnets, as shown in Figure 4. The Dy element is primarily distributed within the main-phase grains because Dy atoms replace the Nd atoms in the 2:14:1 phase, forming the (Nd, Dy)_2_Fe_14_B phase with high magnetic anisotropy, which enhances the magnetic properties of the magnet. The Nd atoms displaced by Dy accumulate at the triangular grain boundaries, forming rare earth-rich phases that act to weaken the exchange coupling. In the 0.2 Ti sample, the distribution of Ti is relatively uniform. However, in the high-Ti samples, Ti elements show significant agglomeration, forming black precipitates. The uneven distribution of Ti black precipitates may be a potential reason for the deterioration in the magnet’s squareness. Due to their low content, these phases were not detected by XRD. The distribution of Ce is relatively uniform and does not accumulate at the triangular grain boundaries like Nd, because the diffused magnets enter the grain boundary phase, which plays a role in dilution.

**Figure 5:**
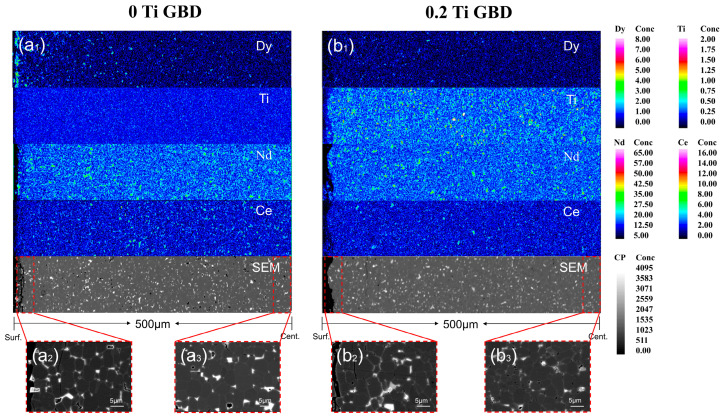
(**a_1_**,**b_1_**) show the EPMA characterization of the 0 Ti GBD and 0.2 Ti GBD samples at 0–500 μm cross-sections; (**a_2_**,**b_2_**) show the SEM images of the 0 Ti GBD and 0.2 Ti GBD samples at the surface; (**a_3_**,**b_3_**) show the SEM images of the 0 Ti GBD and 0.2 Ti GBD samples at 500 μm below the surface.

Figure 5(a_1_,b_1_) show the EPMA results of the 0 Ti and 0.2 Ti samples after grain boundary diffusion at depths from 0 to 500 μm. It can be observed that the concentration of Dy decreases sharply as the diffusion depth increases. In the 0 Ti GBD magnet, the distribution of Dy is uneven, with some of it accumulating on the surface of the magnet and some clustering at the grain boundary region. These two distribution patterns of Dy elements result in inefficient use of Dy, leading to a waste of heavy rare earth resources. In the 0.2 Ti GBD magnet, Dy is distributed more uniformly, with only a small amount of aggregation. This indicates that Ti doping can optimize the distribution of Dy and reduce its clustering at the grain boundaries. Compared to the 0.2 Ti GBD magnet, the reasons for the waste of Dy in the 0 Ti GBD magnet are mainly as follows. First, the discontinuity of the grain boundary phase hinders the diffusion of Dy. As shown in Figure 3, the distribution of the grain boundary phase between the main-phase grains in the 0 Ti magnet is not clear. The grain boundary phase is the primary channel for grain boundary diffusion, and discontinuous grain boundary phases obstruct the internal migration of Dy, causing Dy elements to accumulate largely on the surface of the magnet. Second, the formation of the RE_6_Fe_13_Ga phase promotes the entry of Dy into the main phase. It has been reported [21] that when Dy undergoes grain boundary diffusion, it preferentially enters the main-phase grains rather than the RE_6_Fe_13_Ga phase.

Figure 5(a_2_,a_3_,b_2_,b_3_) represent the BSE images of the 0 Ti GBD and 0.2 Ti GBD samples at the surface and 500 μm below the surface of the magnet. It can be observed that the 0 Ti GBD sample exhibits a distinct shell-like structure on the surface, which almost disappears at 500 μm depth. In contrast, the 0.2 Ti GBD sample shows a relatively thin shell layer on the surface, with the shell-like structure nearly absent at deeper depths. This may be because Dy elements accumulate on the surface of the magnet in the 0 Ti GBD sample, leading to a higher concentration compared to the 0.2 Ti GBD sample. Since diffusion is primarily driven by the concentration gradient, this results in a thicker shell layer for the 0 Ti GBD sample compared to the 0.2 Ti GBD sample. Studies have shown that increasing the thickness of the shell-like structure in magnets can improve their resistance to demagnetization [28]. This may be one of the reasons why the coercivity increase in the 0 Ti GBD magnet is greater than that in the 0.2 Ti GBD magnet.

**Figure 6:**
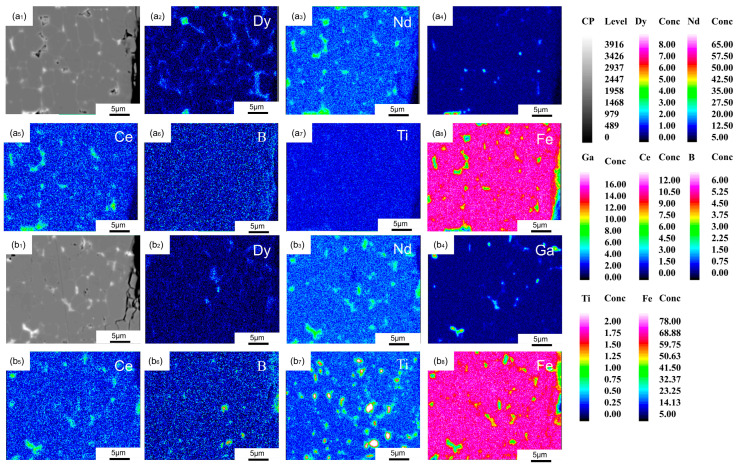
(**a**,**b**) show the EPMA images of the 0 Ti GBD and 0.2 Ti GBD samples at the surface.

To confirm the element distribution differences on the surface of the magnets after diffusion, the specific results are shown in Figure 6. In the 0 Ti GBD magnet, a more distinct shell-like structure can be observed, while the 0.2 Ti GBD magnet shows no noticeable shell structure. In the 0 Ti GBD magnet, significant aggregation of Dy elements is observed at the triangular grain boundary regions. Additionally, in the 0 Ti GBD magnet, Ga elements accumulate in the high Nd, low Fe triangular grain boundary regions, leading to a decrease in the Nd content and an increase in the Fe content in the thin GB regions. Sakuma et al. [29] predicted that the saturation magnetization of the GB phase is directly proportional to the Fe content and inversely proportional to the Nd content. Strong exchange coupling interactions weaken the coercivity of the 0 Ti GBD sample. In the 0.2 Ti GBD magnet, many rare earth-rich phases with higher Ga and Fe content can be observed. This is attributed to the formation of the RE_6_Fe_13_Ga phase [30]. After the addition of Ti, Ti reacts with B to form TiB_2_, thereby reducing the available boron in the alloy. The resulting boron deficiency prevents the formation of the R_2_Fe_14_B phase. Meanwhile, RE and Fe combine with Ga at the grain boundaries, promoting the formation of the RE_6_Fe_13_Ga phase [22].

**Figure 7:**
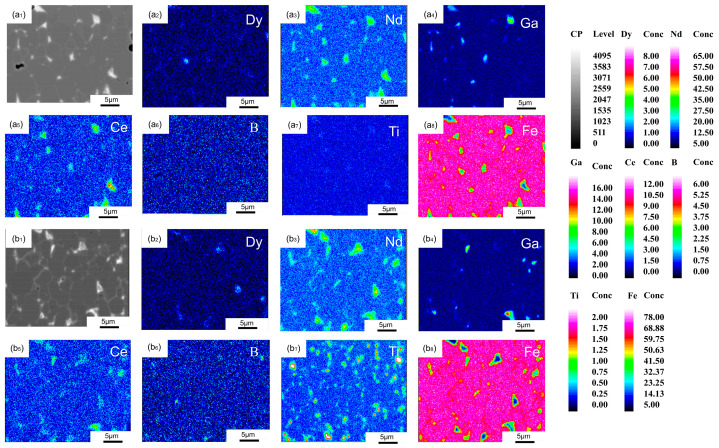
(**a**,**b**) show the EPMA images of the 0 Ti GBD and 0.2 Ti GBD samples at a depth of 500 μm below the surface.

Figure 7 shows the distribution maps of various elements in the 0 Ti GBD and 0.2 Ti GBD samples at a depth of 500 μm below the surface. Neither the 0 Ti GBD nor the 0.2 Ti GBD samples exhibit a distinct shell structure or Dy element aggregation in the triangular grain boundary regions. This is due to the decrease in the Dy element concentration with increasing diffusion depth. In the 0.2 Ti GBD sample, the black precipitates are primarily composed of B and Ti. The distribution of Ga elements is consistent with that observed on the surface of the samples, where Ga elements are mainly distributed in the rare earth-rich triangular grain boundary regions.

**Figure 8:**
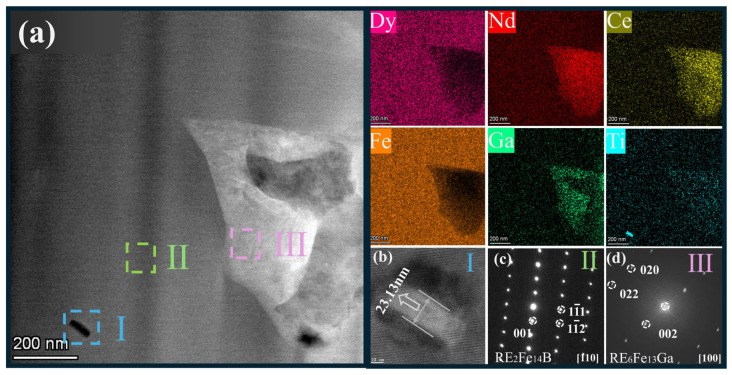
Transmission characterization of the 0.2 Ti GBD sample. (**a**) HAADF; (**b**) High-resolution image of selected region I; (**c**) Selected area electron diffraction (SAED) of selected region II; (**d**) Fourier transform diffraction spots of selected region III.

To further explain the changes in the microstructure of the magnet after Ti addition, Figure 8 shows the TEM spectrum of the 0.2 Ti GBD sample. From Figure 8a, it can be observed that there are black precipitates inside the main-phase grains. By combining with the mapping image, it is evident that the black precipitates are primarily Ti-rich phases. Figure 8b shows a high-resolution image of the selected region I. The width of the black precipitates is found to be 23.13 nm, and no crystalline structure of this phase is observed. Only the lattice fringes of the main phase are visible, as this phase exists inside the main-phase grains. Studies have shown [31] that due to the non-ferromagnetic nature of the TiB_2_ phase, it can suppress the expansion of reverse domains even when it is located inside the grains. Figure 8c shows the diffraction spots corresponding to the lattice structure of RE_2_Fe_14_B in selected region II, which is consistent with the previous XRD results. In Figure 8d, the diffraction spots of selected region III, obtained from the Fourier transform, correspond to the lattice structure of the RE_6_Fe_13_Ga phase, which is consistent with the EPMA and EDS analysis results above. From the mapping image, it can be observed that the Dy element is mainly distributed in the main phase, and there is almost no Dy element in the grain boundary phases. Only a small amount of Dy is present in the RE_6_Fe_13_Ga phase, indicating that Dy elements tend to enter the main phase more readily, which is beneficial for improving the utilization of Dy elements.

The thermal stability of rare earth permanent magnets is typically evaluated using the coercivity temperature coefficient (β) and the remanence temperature coefficient (α) [32,33,34]. Where $Br(T_{1})$, $Br(T_{0})$, $H_{cj}(T_{1})$, and $H_{cj}(T_{0})$ represent the remanence and coercivity of the magnet at temperatures $T_{1}$ and $T_{0}$, respectively.


(2) $$\alpha =\frac{Br\left(T_{1}\right)-Br\left(T_{0}\right)}{Br\left(T_{0}\right)}\times \left(T_{1}-T_{0}\right)\times 100\%$$

(3) $$\beta =\frac{H_{cj}\left(T_{1}\right)-H_{cj}\left(T_{0}\right)}{H_{cj}\left(T_{0}\right)}\times \left(T_{1}-T_{0}\right)\times 100\%$$

**Figure 9:**
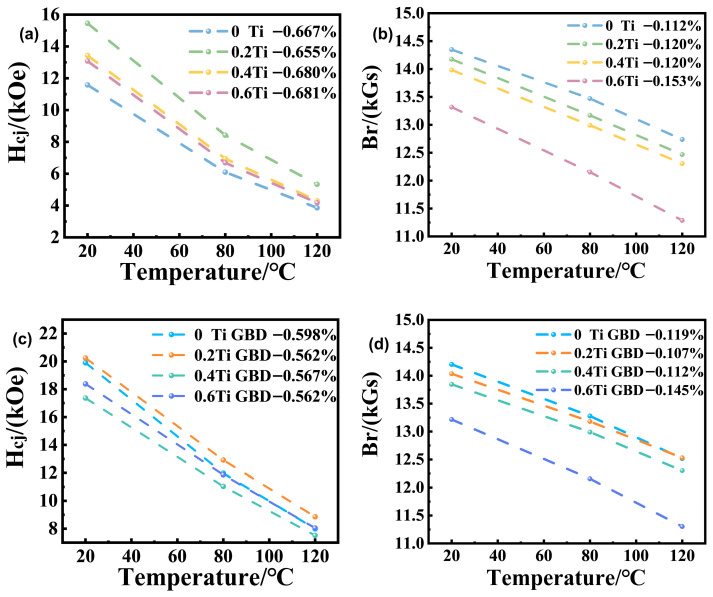
(**a**,**b**) show the variation in coercivity and remanence with temperature from 20 °C to 120 °C for the original magnets; (**c**,**d**) show the variation in coercivity and remanence with temperature from 20 °C to 120 °C for the GBD magnets.

Figure 9 shows the variation in remanence and coercivity with temperature in the range of 20–120 °C for magnets with different Ti contents, both before and after grain boundary diffusion. From Figure 9a, it can be seen that the β value for the 0.2 Ti sample is −0.655%, which indicates better high-temperature coercivity stability compared to the sample without Ti addition (−0.667%). This suggests that the addition of Ti elements helps improve the high-temperature stability of the magnet. However, an excessive amount of Ti elements leads to a decrease in the high-temperature coercivity stability, which is mainly attributed to significant changes in the magnet’s microstructure. As the Ti doping amount increases, the high-temperature stability of the remanence also decreases. From Figure 9c,d, it is observed that after grain boundary diffusion, both the coercivity and remanence stability at high temperatures are improved. This indicates that the diffusion-treated magnets have better thermal stability.

## 4. Conclusions

This study systematically investigates the effect of different Ti doping levels on the Dy grain boundary diffusion behavior in sintered NdFeB magnets and the associated impact on magnetic properties. The experimental results indicate that appropriate Ti doping can significantly optimize the distribution of Dy and improve the overall magnetic performance of the magnet. Microstructural analysis shows that the introduction of Ti promotes the formation of the RE_6_Fe_13_Ga grain boundary phase and increases its volume fraction, thereby enhancing the magnetic isolation of the main-phase grains by the grain boundaries. Additionally, the Ti-B phase formed by Ti combining with B effectively suppresses the abnormal grain growth during sintering, refines the microstructure, and further improves the coercivity of the magnet. The Ti doping alters the distribution behavior of Dy: it facilitates the formation of the RE_6_Fe_13_Ga phase, reduces the excessive retention of Dy in the non-magnetic grain boundary phase, and promotes the effective diffusion of more Dy atoms into the main-phase grain boundaries, contributing to the formation of the hardening shell layer. This mechanism not only improves the coercivity but also enhances the utilization efficiency of the heavy rare earth Dy. Furthermore, the coercivity temperature stability of the Ti-doped samples is improved at high temperatures. In conclusion, this work modulates the distribution of Dy elements through Ti doping, clarifies its mechanism on microstructure and magnetic properties, and provides important theoretical and experimental guidance for the development of high-performance sintered NdFeB magnets with low heavy rare earth content.

## Figures and Tables

**Table 1:** Summary of the properties of original and diffused magnets.

Sample	Br (kGs)		H_cj_ (kOe)		(BH)_max_		Q	
	Original	GBD	Original	GBD	Original	GBD	Original	GBD
0 Ti	14.34	14.14	11.58	19.89	49.14	47.07	0.79	0.62
0.2 Ti	14.17	14.03	15.45	20.24	47.97	47.15	0.98	0.96
0.4 Ti	13.89	13.84	13.43	17.36	45.97	45.40	0.95	0.77
0.6 Ti	13.31	13.21	13.08	18.38	36.73	36.50	0.50	0.40

**Table 2:** EDS Results of Different Locations in the Magnet.

Sample	Position	Pr	Nd	Dy	Ce	Ga	Cu	Fe	Ti
0 Ti	Grain	7.4	19.9	-	2.1	-	-	70.7	-
	White GB	28.3	49.5	-	8.0	1.0	4.6	8.6	-
0.2 Ti	Gray GB	15.1	34.1	-	4.4	4.2	-	42.2	-
	White GB	24.2	43.3	-	8.0	-	1.9	22.6	-
0.4 Ti	Gray GB	14.5	33.7	-	3.8	4.4	-	43.7	-
0 Ti GBD	Grain	6.6	19.8	2.0	1.7	-	-	69.8	-
	White GB	24.2	44.7	1.7	5.7	10.3	2.9	10.4	-
0.2 TiGBD	Grain	6.8	19.9	2.2	1.8	-	-	69.3	-
	White GB	19.7	43.6	4.2	5.6	6.9	3.5	16.6	-
0.4 Ti GBD	Grain	5.4	17.4	6.0	1.9	-	-	69.3	-
	Gray GB	13.7	33.7	-	3.9	4.4	-	44.2	-
0.6 Ti GBD	Abnormal grain	5.3	16.9	3.0	1.8	0.6	0.3	71.4	0.5
	Abnormal grain	5.0	17.1	2.2	1.8	0.7	0.2	72.5	0.6
	Grain	6.1	19.5	2.3	1.7	-	0.1	69.9	0.5

## Data Availability

The original contributions presented in this study are included in the article. Further inquiries can be directed to the corresponding author.
